# 

**Published:** 1880-05

**Authors:** 


CONTENTS.

| | Page | | Page |
|---|---|---|---|
| A Suggestion ............... | 125 | The Mother's Remorse..... | 149 |
| Coughing.................. | 126 | Kindness the Best Punishment ................. | 151 |
| Consumption.............. | 127 | Influence of Christianity on Medical Science........ | 153 |
| Latent Insanity........... | 145 | Constipation ............... | 154 |
| Healthfulness of Fruit..... | 147 | | |
| Health.................... | 148 | | |

